# Risk factor analysis of malignant adenomas detected during colonoscopy

**DOI:** 10.3389/fmed.2023.1106272

**Published:** 2023-02-08

**Authors:** Hong Hu, Xiaoyuan Gong, Kai Xu, Shenzheng Luo, Wei Gao, Baiwen Li, Dadao Jing

**Affiliations:** ^1^Department of Gastroenterology, Shanghai General Hospital, Shanghai Jiao Tong University School of Medicine, Shanghai, China; ^2^Department of General Surgery, Shanghai General Hospital, Shanghai Jiao Tong University School of Medicine, Shanghai, China

**Keywords:** colonoscopy, colorectal adenomas, colorectal cancer, risk factors, screening

## Abstract

**Background:**

Several studies have shown that colorectal adenomas are the most important precancerous lesions. The colonoscopic identification of groups with the high risk of malignant colorectal adenomas remains a controversial issue for clinicians.

**Aims:**

To evaluate the basic characteristics of colorectal adenomas with malignancy risk using high-grade dysplasia (HGD) as an alternative marker for malignant transformation.

**Methods:**

Data from Shanghai General Hospital between January 2017 and December 2021 were retrospectively analyzed. The primary outcome was the incidence of HGD in adenomas, which was used as a surrogate marker for the risk of malignancy. Odds ratios (ORs) for the HGD rate in adenomas were analyzed in relation to adenoma-related factors.

**Results:**

A total of 9,646 patients identified with polyps during 57,445 screening colonoscopies were included in the study. Patients with flat polyps, sessile polyps, and pedunculated polyps represented 27.3% (*N* = 2,638), 42.7% (*N* = 4,114), and 30.0% (*N* = 2,894) of the total number, respectively. HGD was found in 2.41% (*N* = 97), 0.92% (*N* = 24), and 3.51% (*N* = 98) of sessile adenomas, flat adenomas, and pedunculated adenomas, respectively (*P* < 0.001). Multivariable logistic regression showed that polyp size (*P* < 0.001) but not shape (*P* > 0.8), was an independent predictor of HGD. Contrast to the diameter ≤1 cm, the OR value for diameters 1–2, 2–3, and >3 cm was 13.9, 49.3, and 161.6, respectively. The HGD incidence also increased in multiple adenomas (>3 vs. >1, ORs 1.582) and distal adenomas (distal vs. proximal adenomas, OR 2.252). Adenoma morphology (pedunculated vs. flat) was statistically significant in univariate analysis but not when size was included in the multivariate analysis. Besides, the incidence of HGD was also significantly higher in older patients (>64 vs. <50 years old, OR = 2.129). Sex (*P* = 0.681) was not statistically significant. All these associations were statistically significant (*P* < 0.05).

**Conclusion:**

The malignant potential of polyps is mostly affected by their size but not by their shape. In addition, distal location, multiple adenomas, and advanced age were also correlated with malignant transformation.

## Introduction

Colorectal cancer (CRC) is the third most common cancer and fourth most common cause of death globally, accounting for roughly 1.2 million new cases and 600,000 deaths per year. This trend is further increasing as the world grows richer and humans switch to a Western diet. Treatments for CRC are improving, but they are still far from ideal, and identifying and preventing precancerous lesions remains critical. In contrast to sporadic inflammatory and hereditary CRCs, the adenoma-carcinoma pathway underlies the development of most CRCs (1–4). More than 70% of colorectal adenomas progressed to adenomatous carcinoma through a series of gene mutations. Adenomas are considered precursors in most cases of CRC (5). Patients with advanced adenoma are significantly more likely to develop CRC and are at a significantly increased risk of CRC death compared to patients without adenoma (6–8).

Endoscopy is still the most significant examination for the prevention and detection of early colon cancer because it can detect the size, shape, location, and activity of tumors and can take a biopsy of suspicious lesions under a directional microscope (9). When endoscopists perform colonoscopy, the early identification of high-risk adenomas and high-risk groups is of great significance for the selection of treatment methods and follow-up time.

This study aimed to evaluate the effect of the shape, size, location, and number of endoscopically detected adenomas on malignant transformation based on the adenoma-carcinoma progression hypothesis, using high-grade dysplasia (HGD) as a surrogate marker for CRC, combined with age and sex distribution, to provide a theoretical basis for early identification of high-risk adenomas.

## Materials and methods

### Patients

**FIGURE 1:**
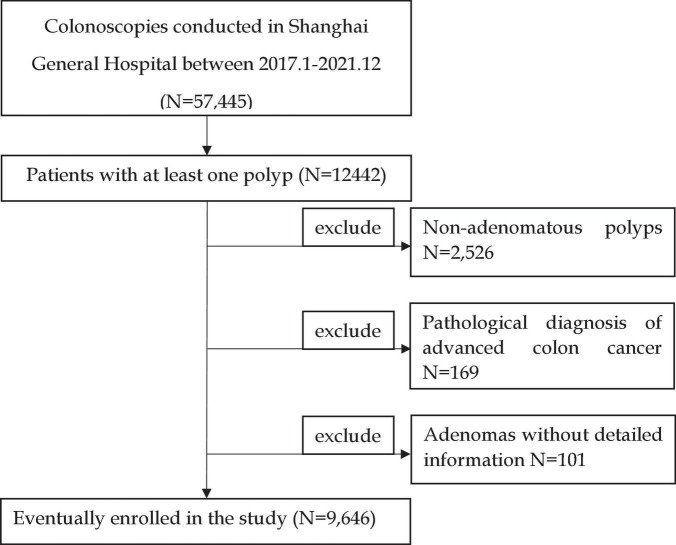
Flowchart of the patients included in the study.

We recorded the results of colonoscopies performed at the Shanghai General Hospital from January 2017 to December 2021. A total of 57,445 colonoscopies were documented, of which 12,442 detected polyps. A total of 2,526 pathologically suggested non-adenomatous polyps, 169 patients diagnosed with colon cancer, and 101 patients without detailed information records were excluded. The inclusion and exclusion criteria are shown in Figure 1. Finally, 9,646 patients were included in the study. If more than one polyp was found, only the adenoma with the most advanced histology or the largest polyp was recorded in detail as the target adenoma.

#### Documented data used for this analysis are as follows

**FIGURE 2:**
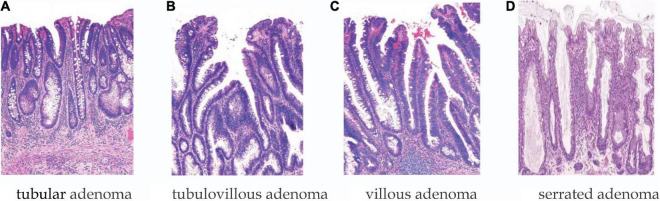
Histology: **(A)** Tubular, **(B)** tubulovillous, **(C)** villous, and **(D)** serrated adenomas.

Patients were men and women divided into age groups of <50 years, 50–64 years, and ≥65 years. The number of polyps was divided into the following categories: 1, 2–3, and >3; polyp size was distinguished according to the following categories: <1, 1–2, 2–3, and >3 cm. Shape: pedunculated/sessile/flat. Lesion morphology was classified according to the Paris classification: pedunculated (Paris Ip), sessile (Paris Is), and flat (Paris IIa, IIb, and IIc). Location categories included distal locations (i.e., descending colon, rectum, and sigmoid colon) and proximal locations (above the descending colon). Histology (shown in Figure 2): tubular, villous, tubulovillous, serrated adenomas, and the category of HGD, with the latter including carcinoma *in situ* in accordance with the World Health Organization definition.

### Statistical analysis

All statistical tests were conducted using SPSS version 26.0 (IBM Corporation, Armonk, NY, USA). Continuous variables were expressed as mean ± standard deviation (±SD) or median ± interquartile range (Md ± IQR). Normally distributed data were analyzed using a 2-tailed *t*-test, whereas non-normally distributed data were analyzed using the Mann–Whitney *U*-test. Categorical variables are indicated as proportions and analyzed using the χ^2^ test. If >20% of the expected value was less than 5, Fisher’s exact test was used. To control for potential confounding between predictor variables, binary logistic regression was performed to calculate the odds ratios (ORs) with 95% confidence intervals (CIs). Statistical significance was defined as *P* < 0.05.

## Results

### Patient and polyp characteristics

Details of the included patients and the adenomas detected are shown in Table 1. In total, 9,646 patients with adenomas were identified. The adenomas were ≤5 mm in size in 29.3% of cases, and only 22.6% were >1 cm. HGD was found in 2.3% of adenomas (*N* = 219).

**TABLE 1:** Basic characteristics of patient and polyp.

Characteristic	Study population (*N* = 9,646)	
Patient age, mean (SD), range	57.35 (11.827)	17–92
Patient sex, male: female (%)	Male 6,205, 64.3%	Female 3,441, 35.7%
	*N*	%
**Adenoma size**		
<0.5 cm	2,827	29.3
0.5–1 cm	4,634	48.0
1–1.5 cm	1,321	13.7
1.5–2 cm	485	5.0
2–3 cm	260	2.7
>3 cm	119	1.2
**Adenoma shape**		
Pedunculated	2,894	30.0
Sessile	4,114	42.7
Flat	2,638	27.3
**Adenoma histology**		
Tubular	8,877	92.0
Tubulovillous	304	3.2
Villous	15	0.2
Serrated	231	2.4
HGD	219	2.3
**Adenoma location**		
Proximal	4,240	44.0
Distal	5,406	56.0

Adenomas reported are target adenomas (i.e., those with the most severe histology or maximum size). HGD, high-grade dysplasia. Distal = descending colon, rectum, and sigmoid colon; proximal = above the descending colon.

### Univariate and multivariate analysis of risk factors for high-grade dysplasia in adenomas

#### Univariate analysis

According to the presence or absence of HGD, 9,646 patients with adenoma were divided into the adenoma group (9,427 cases) and the HGD group (219 cases). There was no difference in sex (*P* = 0.681) between the two groups; however, there were significant differences in age, adenoma location, adenoma number, adenoma morphology, and adenoma size (*P* < 0.001), as shown in Table 2.

**TABLE 2:** Univariate analysis of adenoma factors relative to the occurrence of HGD.

	Adenoma (*N* = 9,427)	HGD (*N* = 219)		*P*-value
	***N* (%)**	***N* (%)**		
Sex			*X*^2^ = 0.168	0.681
Male	6,067 (62.9)	138 (1.4)		
Female	3,360 (34.8)	81 (0.8)		
Age			*X*^2^ = 27.192	<0.001
<50	2,320 (24.1)	24 (0.3)		
50–64	4,266 (44.2)	102 (1.1)		
≥65	2,934 (30.4)	93 (1.0)		
Size			*X*^2^ = 1,275.171	<0.001
≤1 cm	7,435 (77.1)	26 (0.3)		
1–2 cm	1,708 (17.7)	98 (1.0)		
2–3 cm	213 (2.2)	47 (0.5)		
>3 cm	71 (0.7)	48 (0.5)		
Amount			*X*^2^ = 58.958	<0.001
1	5,187 (53.8)	73 (0.8)		
2–3	2,622 (27.2)	69 (0.7)		
>3	1,618 (16.8)	77 (0.8)		
Morphology			*X*^2^ = 38.394	<0.001
Sessile	4,017 (41.6)	97 (1.0)		
Flat	2,614 (27.1)	24 (0.2)		
Pedunculated	2,796 (29.0)	98 (1.0)		
Location			*X*^2^ = 46.030	<0.001
Proximal	4,193 (43.5)	47 (0.5)		
Distal	5,234 (54.3)	172 (1.8)		

HGD, high-grade dysplasia. Distal = descending colon, rectum, and sigmoid colon; proximal = above the descending colon.

#### Multivariate analysis

The Table 3 shows the percentages of adenoma sizes and HGD with different morphologies. Table 3 also shows the distribution of HGD in adenomas of different morphologies: flat, sessile, or pedunculated. The overall risk of HGD diagnosis in patients with pedunculated lesions was 3.39% (*N* = 98), compared to 2.41% (*N* = 97) in patients with sessile lesions and 0.92% in patients with flat lesions (*P* < 0.05).

**TABLE 3:** Size distribution of adenoma shape.

Polyp	Polyp shape								
	**Pedunculated**			**Sessile**			**Flat**		
	***N***	**Of those, HGDs**	**HGD (%)**	***N***	**Of those, HGDs**	**HGD (%)**	***N***	**Of those, HGDs**	**HGD (%)**
**Size**									
<0.5	256	0	0.00	1,254	1	0.08	1,317	1	0.08
0.5–1	1,507	11	0.73	1,982	8	0.40	1,145	5	0.44
1–1.5	676	22	3.25	521	22	4.22	124	2	1.61
1.5–2	276	30	10.87	178	18	10.11	31	4	12.90
2–3	139	25	17.99	111	18	16.22	10	4	40.0
>3	40	10	25.00	68	30	44.12	11	8	72.73
All cases	2,894	98	3.39	4,017	97	2.41	2,614	24	0.92

HGD, high-grade dysplasia. Distal = descending colon, rectum, and sigmoid colon; proximal = above the descending colon.

In addition, there was a significant difference in the prevalence of HGD in different sizes of the three types of adenomas. Table 3 shows that adenomas ≤10 mm were less likely to develop HGD regardless of whether they were pedunculated or sessile adenomas (≤1 cm, pedunculated vs. sessile vs. flat, 0.73% vs. 0.48% vs. 0.52%, *P* < 0.05).

To confirm these findings, we performed multivariable logistic regression analyses for polyp size and shape with additional adjustments for age, number of adenomas, and adenoma location. Regression analysis showed that polyp size, age, and location were statistically significant independent risk factors for HGD (*P* < 0.001).

Polyp shape was a statistically significant risk factor for HGD in the univariate model (*P* < 0.0001). However, polyp shape was no longer a statistical risk factor for HGD when polyp size was included in the multivariate model (*P* > 0.8), as detailed in Table 4.

**TABLE 4:** Comparison of univariate and multivariate analysis of adenoma factors relative to the occurrence of HGD.

	Univariate	Multivariate
**Size**		
*P*	<0.001	<0.001
**OR (95% CI)**		
≤1 cm	1	
1–2 cm	16.408 (10.614–25.363)	13.890 (8.756–22.034)
2–3 cm	63.099 (38.347–103.829)	48.684 (28.641–82.755)
>3 cm	193.326 (113.632–328.913)	165.599 (95.244–287.923)
**Morphology**		
*P*	<0.001	>0.800
**OR (95% CI)**		
Sessile	2.630 (1.678–4.123)	0.957 (0.580–1.579)
Pedunculated	3.818 (2.435–5.984)	0.946 (0.568–1.575)
Flat	1	
**Amount**		
*P*	<0.001	<0.05
**OR (95% CI)**		
1	1	
2–3	2.131 (1.559–2.914)	1.261 (0.893–1.781)
>3	3.182 (2.238–4.523)	1.582 (1.070–2.339)
**Age**		
*P*	<0.001	<0.05
**OR (95% CI)**		
<50	1	
50–64	2.311 (1.477–3.166)	1.866 (1.152–3.022)
>64	3.164 (2.014–4.973)	2.129 (1.300–3.487)
**Location**		
*P*	<0.001	<0.001
**OR (95% CI)**		
Proximal	1	
Distal	2.932 (2.118–4.059)	2.252 (1.589–3.190)

HGD, high-grade dysplasia, CI, confidence interval. Distal = descending colon, rectum, and sigmoid colon; proximal = above the descending colon.

### Age distribution

Table 5 shows the age distribution of the adenomas. The patients were divided into two groups: <60 years and ≥60 years. Compared with non-elderly patients, elderly patients had a higher proportion of proximal adenomas (47.26% vs. 41.32%), multiple adenomas (54.26% vs. 38.45%), more macroadenomas (25.82% vs. 20.12%), and a higher malignant transformation rate than young patients (3.11% vs. 1.60%). The morphological distribution was not significantly worse (pedunculated vs. sessile, 29.89% vs. 30.10%), all of which were statistically significant (*P* < 0.05).

**TABLE 5:** Age distribution of adenoma location, amount, size, and shape.

	<60 years old	Of those, HGD (%)	≥60 years old	Of those, HGD (%)
Location	<0.001			
Proximal	2,216, 41.32%	16, 0.72	2,024, 47.26%	31, 1.53
Distal	3,147, 58.68%	70, 2.22	2,259, 52.74%	102, 4.52
Amount	<0.001			
1	3,301, 61.55%	36, 1.09	1,959, 45.74%	37, 1.89
>1	2,062, 38.45%	50, 2.42	2,324, 54.26%	96, 4.13
Size	<0.001			
≤1 cm	4,284, 79.88%	11, 0.26	3,177, 74.18%	15, 0.47
1–2 cm	913, 17.02%	41, 4.49	893, 20.85%	57, 6.38
2–3 cm	118, 2.20%	18, 15.25	142, 3.32%	29, 20.42
>3 cm	48, 0.90%	16, 33.33	71, 1.66%	32, 45.07
Morphology	<0.001			
Pedunculated	1,614	46, 2.85	1,280	52, 4.06
Sessile	3,749	40, 1.07	3,003	81, 2.70
Flat	5,363	86, 1.60	4,283	133, 3.11

### Sex distribution

Table 6 shows the sex distribution of the adenomas. The incidence of pedunculated adenoma was higher in men than in women (31.01% vs. 28.19%, *P* < 0.05), but the incidence of distal adenoma was lower (54.25% vs. 59.29%, *P* < 0.05). However, there was no significant difference in the incidence of HGD between men and women (2.35% vs. 2.22%, *P* = 0.681).

**TABLE 6:** Sex distribution of adenoma location and shape.

	All cases (mean age, 57.35 years)		Men (mean age, 56.79 years)		Women (mean age, 58.35 years)	
	***N***	**%**	***N***	**%**	***N***	**%**
**All patients with adenomas**	**9,646**	**100%**	**6,205**	**64.3%**	**3,441**	**35.7%**
**Adenoma location**						
Proximal	4,240	44.0	2,839	45.75	1,401	40.71
Of those, HGD	47	1.11	28	0.99	29	2.07
Distal	5,406	56.0	3,366	54.25	2,040	59.29
Of those, HGD	172	3.18	110	3.27	62	3.04
**Adenoma shape**						
Pedunculated	2,894	30.00	1,924	31.01	970	28.19
Of those, HGD	98	3.39	67	3.48	31	3.20
Sessile	6,752	70.00	4,281	68.99	2,471	71.81
Of those, HGD	121	1.79	71	1.66	50	2.02
**Adenoma shape and location**						
Proximal pedunculated	1,178	12.21	794	12.80	384	11.16
Of those, HGD	17	1.44	10	1.26	7	1.82
Distal pedunculated	1,716	17.79	1,130	18.21	586	17.03
Of those, HGD	81	6.88	57	5.04	24	4.10
Proximal sessile	3,062	31.74	2,045	32.96	1,017	29.56
Of those, HGD	30	0.98	18	0.88	12	1.18
Distal sessile	3,690	38.25	2,236	36.04	1,454	42.26
Of those, HGD	91	2.47	53	2.37	35	2.41

HGD, high-grade dysplasia. Distal = descending, rectum, and sigmoid colon; proximal = above the descending colon.

## Discussion

The identification of groups at high risk of colorectal adenoma remains a controversial issue for clinicians.

The use of HGD as a surrogate marker for the risk of cancer development from adenomas seems to be accepted, based on the concept of the adenoma-carcinoma sequence, although it is not fully known how long HGD persists before it develops into carcinoma or to what extent this is related to other risk factors (10–12). There is evidence of an increased risk of cancer development from HGD in the upper gastrointestinal tract (13). Besides, HGD is also associated with an increased risk of colon cancer in patients with inflammatory bowel disease (14, 15).

Research on the location of adenoma and the risk of cancer has also been controversial. The question of the “biology” of the left colon vs. the right colon has puzzled many scholars. Recent retrospective analyses have noted that a significantly smaller volume but a proximal location of proximal adenomas is associated with a higher incidence of malignancy (16–19). In addition, CRC mortality after polypectomy was lower in patients with right-sided adenomas in the Norwegian Cancer Registry (12, 20). There is also a significant difference in the location of adenoma between the elderly and the young. Statistical data show that the incidence of colorectal tumors in the young has increased year by year in recent years, and the main incidence is concentrated in the left colon and rectum (21). At present, advanced colon cancer has entered the era of precision treatment under the guidance of the primary site (left and right colon). Solving the problem of the location of colorectal adenoma is of guiding significance for precision treatment. HGD was significantly more common in distal adenomas than in proximal adenomas in this study. The location of the adenoma does not fully account for this contradiction (22, 23). This may be partly due to the earlier appearance of clinical symptoms such as blood in the stool and changes in stool shape and bowel habits in patients with distant adenomas, which prompt people to seek more medical advice (24).

The role of adenoma shape has been debated for many years. Some studies have shown that sessile lesions have a higher risk of malignancy (25, 26); however, there is also evidence to support the higher HGD rate of pedunculated adenomas (27). In the present study, the incidence of HGD was higher in pedunculated adenomas than in flat adenomas in the univariate analysis. This is partly due to the higher proportion of large pedunculated adenomas than flat adenomas (>1 cm, pedicled vs. flat, 39.08% vs. 6.81%). However, this difference was lost when size was included in the multivariate analysis, which is consistent with the findings of Reinhart et al. (28). The influence of adenoma morphology is still controversial, but our results suggest that it is not an independent risk factor for malignant transformation of adenomas. The Paris classification was used for adenoma morphology in this paper, but no morphological significance could be observed. More detailed morphological classification further studies may be needed to confirm this conclusion. Some studies have suggested that villous components are closely related to the malignant potential of adenomas, but whether this is also affected by the factor of adenoma size is unknown. We cannot verify this point due to the small number of villous adenoma samples in this study. We look forward to further studies to analyze the role of villous components in adenomas of similar size in the future.

Other risk factors such as adenoma size and patient age were confirmed in this study. Both large size and advanced age were positively correlated with HGD (29). In this study, compared with adenomas <1 cm, the OR for polyps 2 cm and 2–3 cm were 13.890 (8.756, 22.034) and 48.684 (28.641, 82.755), respectively, and the OR for polyps >3 cm was 165.599 (95.244, 287.923). The large CIs were due to the low total number of HGDs. However, a high OR clearly indicated the effect of size on HGD incidence. The effect of size on the prevalence of advanced cancer was consistent with the data from other studies. Nearly all studies reported a risk of severe dysplasia of less than 1% in small (<10 mm) adenomas, and our results fall within this range (0.59%) (27, 28).

Related studies have shown that the recurrence rate of multiple adenomas after colonoscopic resection is significantly higher than that of single adenomas, and adenoma recurrence is considered to be one of the main risk factors for malignant transformation. At the same time, our analysis found that the incidence of HGD in patients with multiple adenomas was significantly higher than those with single adenomas (>3 vs. 1, OR 1.582) (30–32). Patients with familial adenomatous polyposis have a high rate of malignant transformation, and we speculate that patients with multiple polyps may have a higher genetic susceptibility (33, 34).

Many studies have shown that age is associated with the development of CRC (35). Some studies have found that patients <50 years of age are more likely to have distal CRC, while older patients are more likely to have proximal CRC (36). In this study, the elderly and non-elderly groups were divided using 60 years as the baseline, and there was no significant difference in the morphological distribution between the two groups (pedicled vs. sessile, 29.89% vs. 30.10%). In the elderly group, the proportion of proximal adenomas was higher (> 60 vs. ≤60 years, 47.26% vs. 41.32%), multiple adenomas were more common (> 60 vs. ≤60 years, 54.26% vs. 38.45%), and large adenomas >1 cm were more frequent (>60 vs. ≤60 years, 25.82% vs. 20.12%). The rate of HGD in the elderly group was higher than that in the non-elderly group (>60 vs. ≤60 years, 3.11% vs. 1.60%), and the OR value was 1.539 (95% CI: 1.139–2.080). Our results suggest that adenomas in elderly patients had more features of high-risk, and more active treatment measures should be taken in patients with adenomas >60 years.

In some studies, men and women had different risks of CRC, which may be related to smoking, alcohol consumption, obesity, and other factors (37–39). No effect of sex difference on HGD incidence was observed in the present study, which is in line with the findings of Rösch et al. (27).

This study had some limitations. (1) This was a retrospective study, and there was a selection and information biases in the data collection process due to the possibility of convenient sampling and incomplete or missing patient records. (2) The sample population selection and construction process were all conducted in the same medical institution, which was a single-center study with certain limitations. In the future, a multicentre study should be conducted for further verification. (3) Data from only one adenoma per patient, the one that was most important in terms of histology or size, were analyzed. This may have introduced some bias, particularly in patients with multiple polyps, which may have diluted some of the observed effects.

## Conclusion

In conclusion, this study shows that when HGD is used as a surrogate marker for CRC, the effect of sex and morphology on malignancy is controversial, but adenoma size is the most important factor in the development of HGD in all morphologic adenomas. Adenomas detected in the distal colon had a higher incidence of HGD than those detected in the proximal colon. Patients with multiple adenomas have a higher incidence of HGD. Adenomas in elderly patients had more features of high-risk, more active treatment and follow-up should be performed in patients with high-risk adenomas.

## Data availability statement

The original contributions presented in this study are included in the article/supplementary material, further inquiries can be directed to the corresponding author.

## Ethics statement

The studies involving human participants were reviewed and approved by the Ethics Committee of Shanghai General Hospital, Shanghai Jiao Tong University School of Medicine (IRB No. 021KY049). The patients/participants provided their written informed consent to participate in this study. The respondents were informed about the aim of the study. This study was conducted in accordance with the declaration of Helsinki. The confidentiality and anonymity of the data was also ensured.

## Author contributions

All authors made a significant contribution to the work reported, whether that is in the conception, study design, execution, acquisition of data, analysis and interpretation, or in all these areas, took part in drafting and revising or critically reviewing the article, gave final approval of the version to be published, have agreed on the journal to which the article has been submitted, and agreed to be accountable for all aspects of the work.

## References

[1] Brenner H Kloor M Pox CP. Colorectal cancer. *Lancet.* (2014) 383:1490–502. 10.1016/S0140-6736(13)61649-9 24225001

[2] Weitz J Koch M Debus J Höhler T Galle PR Büchler MW. Colorectal cancer. *Lancet.* (2005) 365:153–65. 15639298 10.1016/S0140-6736(05)17706-X

[3] Brody H. Colorectal cancer. *Nature.* (2015) 521:S1. 10.1038/521S1a 25970450

[4] Dekker E Tanis PJ Vleugels JL Kasi PM Wallace MB. Colorectal cancer. *Lancet.* (2019) 394:1467–80. 10.1016/S0140-6736(19)32319-0 31631858

[5] Komor MA Bosch LJ Bounova G Bolijn AS Diemen PM Rausch C Consensus molecular subtype classification of colorectal adenomas. *J Pathol.* (2018) 246:266–76. 29968252 10.1002/path.5129 PMC6221003

[6] Click B Pinsky PF Hickey T Doroudi M Schoen RE. Association of colonoscopy adenoma findings with long-term colorectal cancer incidence. *JAMA.* (2018) 319:2021–31. 10.1001/jama.2018.5809 29800214 PMC6583246

[7] Miller EA Pinsky PF Schoen RE Prorok PC Church TR. Effect of flexible sigmoidoscopy screening on colorectal cancer incidence and mortality: long-term follow-up of the randomised US PLCO cancer screening trial. *Lancet Gastroenterol Hepatol.* (2019) 4:101–10. 10.1016/S2468-1253(18)30358-3 30502933 PMC6335177

[8] Cottet V Jooste V Fournel I Bouvier A Faivre J Bonithon-Kopp C. Long-term risk of colorectal cancer after adenoma removal: a population-based cohort study. *Gut.* (2012) 61:1180–6. 10.1136/gutjnl-2011-300295 22110052

[9] Lin JS Piper MA Perdue LA Rutter CM Webber EM Connor E Screening for colorectal cancer: updated evidence report and systematic review for the US preventive services task force. *JAMA.* (2016) 315:2576–94. 10.1001/jama.2016.3332 27305422 PMC13623242

[10] Leslie A Carey FA Pratt NR Steele RJ. The colorectal adenoma-carcinoma sequence. *Br J Surg.* (2002) 89:845–60. 10.1046/j.1365-2168.2002.02120.x 12081733

[11] Nakamura F Sato Y Okamoto K Fujino Y Mitsui Y Kagemoto K Colorectal carcinoma occurring via the adenoma-carcinoma pathway in patients with serrated polyposis syndrome. *J Gastroenterol.* (2022) 57:286–99. 10.1007/s00535-022-01858-8 35194694

[12] Calderwood AH Lasser KE Roy HK. Colon adenoma features and their impact on risk of future advanced adenomas and colorectal cancer. *World J Gastrointest Oncol.* (2016) 8:826–34. 10.4251/wjgo.v8.i12.826 28035253 PMC5156849

[13] Conio M Cameron AJ Chak A Blanchi S Filiberti R. Endoscopic treatment of high-grade dysplasia and early cancer in Barrett’s oesophagus. *Lancet Oncol.* (2005) 6:311–21. 10.1016/S1470-2045(05)70167-4 15863379

[14] Pulusu SS Lawrance IC. Dysplasia and colorectal cancer surveillance in inflammatory bowel disease. *Expert Rev Gastroenterol Hepatol.* (2017) 11:711–22. 10.1080/17474124.2017.1327347 28475382

[15] Murthy SK Feuerstein JD Nguyen GC Velayos F. AGA clinical practice update on endoscopic surveillance and management of colorectal dysplasia in inflammatory bowel diseases: expert review. *Gastroenterology.* (2021) 161:1043–51.e4. 10.1053/j.gastro.2021.05.063 34416977

[16] Sawhney MS Dickstein J LeClair J Lembo C Yee E. Adenomas with high-grade dysplasia and early adenocarcinoma are more likely to be sessile in the proximal colon. *Colorectal Dis.* (2015) 17:682–8. 10.1111/codi.12911 25619115

[17] Benedix F Kube R Meyer F Schmidt U Gastinger I Lippert H Comparison of 17,641 patients with right- and left-sided colon cancer: differences in epidemiology, perioperative course, histology, and survival. *Dis Colon Rectum.* (2010) 53:57–64. 10.1007/DCR.0b013e3181c703a4 20010352

[18] Klein JL Okcu M Preisegger KH Hammer HF. Distribution, size and shape of colorectal adenomas as determined by a colonoscopist with a high lesion detection rate: influence of age, sex and colonoscopy indication. *United European Gastroenterol J.* (2016) 4:438–48. 10.1177/2050640615610266 27403311 PMC4924432

[19] Loree JM Pereira AA Lam M Willauer AN Raghav K Dasari A Classifying colorectal cancer by Tumor location rather than sidedness highlights a continuum in mutation profiles and consensus molecular subtypes. *Clin Cancer Res.* (2018) 24:1062–72. 10.1158/1078-0432.CCR-17-2484 29180604 PMC5844818

[20] de Jonge V Nicolaas JS Leerdam ME Kuipers EJ Zanten SJ. Systematic literature review and pooled analyses of risk factors for finding adenomas at surveillance colonoscopy. *Endoscopy.* (2011) 43:560–72. 10.1055/s-0030-1256306 21437854

[21] Ouakrim DA Pizot C Boniol M Malvezzi M Boniol M Negri E Trends in colorectal cancer mortality in Europe: retrospective analysis of the WHO mortality database. *BMJ.* (2015) 351:h4970. 10.1136/bmj.h4970 26442928 PMC4595561

[22] Xiang L Zhan Q Zhao X Wang Y An S Xu Y Risk factors associated with missed colorectal flat adenoma: a multicenter retrospective tandem colonoscopy study. *World J Gastroenterol.* (2014) 20:10927–37. 10.3748/wjg.v20.i31.10927 25152596 PMC4138473

[23] Xiang L Zhan Q Wang X Zhao X Zhou Y An S Risk factors associated with the detection and missed diagnosis of colorectal flat adenoma: a Chinese multicenter observational study. *Scand J Gastroenterol.* (2018) 53:1519–25. 10.1080/00365521.2018.1533581 30621477

[24] Nawa T Kato J Kawamoto H Okada H Yamamoto H Kohno H Differences between right- and left-sided colon cancer in patient characteristics, cancer morphology and histology. *J Gastroenterol Hepatol.* (2008) 23:418–23. 17532785 10.1111/j.1440-1746.2007.04923.x

[25] Rembacken BJ Fujii T Cairns A Dixon MF Yoshida S Chalmers DM Flat and depressed colonic neoplasms: a prospective study of 1000 colonoscopies in the UK. *Lancet.* (2000) 355:1211–4. 10770302 10.1016/s0140-6736(00)02086-9

[26] Hart AR Kudo S Mackay EH Mayberry JF Atkin WS. Flat adenomas exist in asymptomatic people: important implications for colorectal cancer screening programmes. *Gut.* (1998) 43:229–31. 10189849 10.1136/gut.43.2.229 PMC1727201

[27] Rösch T Altenhofen L Kretschmann J Hagen B Brenner H Pox C Risk of malignancy in adenomas detected during screening colonoscopy. *Clin Gastroenterol Hepatol.* (2018) 16:1754–61. 29902640 10.1016/j.cgh.2018.05.043

[28] Reinhart K Bannert C Dunkler D Salzl P Trauner M Renner F Prevalence of flat lesions in a large screening population and their role in colonoscopy quality improvement. *Endoscopy.* (2013) 45:350–6. 23616125 10.1055/s-0032-1326348

[29] Pommergaard H Burcharth J Rosenberg J Raskov H. The association between location, age and advanced colorectal adenoma characteristics: a propensity-matched analysis. *Scand J Gastroenterol.* (2017) 52:1–4. 10.1080/00365521.2016.1218929 27686516

[30] Facciorusso A Maso MD Serviddio G Vendemiale G Muscatiello N. Development and validation of a risk score for advanced colorectal adenoma recurrence after endoscopic resection. *World J Gastroenterol.* (2016) 22:6049–56. 10.3748/wjg.v22.i26.6049 27468196 PMC4948260

[31] Bonithon-Kopp C Piard F Fenger C Cabeza E Morain C Kronborg O Colorectal adenoma characteristics as predictors of recurrence. *Dis Colon Rectum.* (2004) 47:323–33. 14991494 10.1007/s10350-003-0054-1

[32] Nusko G Hahn EG Mansmann U. Characteristics of metachronous colorectal adenomas found during long-term follow-up: analysis of four subsequent generations of adenoma recurrence. *Scand J Gastroenterol.* (2009) 44:736–44. 19277927 10.1080/00365520902770078

[33] Galiatsatos P Foulkes WD. Familial adenomatous polyposis. *Am J Gastroenterol.* (2006) 101:385–98. 16454848 10.1111/j.1572-0241.2006.00375.x

[34] Half E Bercovich D Rozen P. Familial adenomatous polyposis. *Orphanet J Rare Dis.* (2009) 4:22. 10.1186/1750-1172-4-22 PMC2772987 19822006

[35] Laish I Mizrahi J Naftali T Konikoff FM. Diabetes mellitus and age are risk factors of interval colon cancer: a case-control study. *Dig Dis.* (2019) 37:291–6. 30731459 10.1159/000496740

[36] Weinberg BA Marshall JL. Colon cancer in young adults: trends and their implications. *Curr Oncol Rep.* (2019) 21:3. 10.1007/s11912-019-0756-8 30659375

[37] Cho S Shin A. Population attributable fraction of established modifiable risk factors on colorectal cancer in Korea. *Cancer Res Treat.* (2021) 53:480–6. 10.4143/crt.2019.742 33070559 PMC8053879

[38] Jodal HC Klotz D Herfindal M Barua I Tag P Helsingen LM Long-term colorectal cancer incidence and mortality after adenoma removal in women and men. *Aliment Pharmacol Ther.* (2022) 55:412–21. 34716941 10.1111/apt.16686

[39] Schmuck R Gerken M Teegen E Krebs I Klinkhammer-Schalke M Aigner F Gender comparison of clinical, histopathological, therapeutic and outcome factors in 185,967 colon cancer patients. *Langenbecks Arch Surg.* (2020) 405:71–80. 10.1007/s00423-019-01850-6 32002628 PMC7036075

